# Genetic Factors That Might Lead to Different Responses in Individuals Exposed to Perchlorate

**DOI:** 10.1289/ehp.8076

**Published:** 2005-06-29

**Authors:** Franco Scinicariello, H. Edward Murray, Lester Smith, Sharon Wilbur, Bruce A. Fowler

**Affiliations:** Division of Toxicology, Agency for Toxic Substances and Disease Registry, Centers for Disease Control and Prevention, Atlanta, Georgia, USA

**Keywords:** genetic susceptibility, hypothyroidism, mutations, NIS, Pendred syndrome, pendrin, perchlorate, thyroid gland, TPO

## Abstract

Perchlorate has been detected in groundwater in many parts of the United States, and recent detection in vegetable and dairy food products indicates that contamination by perchlorate is more widespread than previously thought. Perchlorate is a competitive inhibitor of the sodium iodide symporter, the thyroid cell–surface protein responsible for transporting iodide from the plasma into the thyroid. An estimated 4.3% of the U.S. population is subclinically hypothyroid, and 6.9% of pregnant women may have low iodine intake. Congenital hypothyroidism affects 1 in 3,000 to 1 in 4,000 infants, and 15% of these cases have been attributed to genetic defects. Our objective in this review is to identify genetic biomarkers that would help define subpopulations sensitive to environmental perchlorate exposure. We review the literature to identify genetic defects involved in the iodination process of the thyroid hormone synthesis, particularly defects in iodide transport from circulation into the thyroid cell, defects in iodide transport from the thyroid cell to the follicular lumen (Pendred syndrome), and defects of iodide organification. Furthermore, we summarize relevant studies of perchlorate in humans. Because of perchlorate inhibition of iodide uptake, it is biologically plausible that chronic ingestion of perchlorate through contaminated sources may cause some degree of iodine discharge in populations that are genetically susceptible to defects in the iodination process of the thyroid hormone synthesis, thus deteriorating their conditions. We conclude that future studies linking human disease and environmental perchlorate exposure should consider the genetic makeup of the participants, actual perchlorate exposure levels, and individual iodine intake/excretion levels.

Sequencing of the human genome has brought new emphasis and increased interest in gene–environment interactions and is becoming relevant in defining public health policies. For many years, people’s susceptibility to xenobiotics have been known to differ significantly. Now, several techniques are available to identify and characterize the genetic correlates of interindividual variability. The goal of environmental genomics is to help investigators understand how genetic variability influences individual responses to environmental factors on the basis of the assumption that high-risk genotypes accumulate more damage and therefore are at greater risk of developing exposure-related diseases. Thus, genomics information may lead to development of predictive biomarkers that identify potentially sensitive populations and earlier prediction of adverse outcomes, ultimately resulting in better intervention strategies (Kelada et al. 2003).

## Public Health and Perchlorate

The advent and use of new, highly sensitive detection techniques have identified contamination of groundwater by perchlorate in many parts of the United States, primarily in association with industries involved in rocket, explosives, and fireworks manufacturing and propellant handling. Concentrations measured in most public water supplies are < 50 μg/L; however, levels as high as several hundred microliters per liter have been reported in some drinking water wells in certain communities (Motzer 2001). The recent detection of perchlorate in vegetable and dairy food products (Kirk et al. 2003; Smith et al. 2001) indicates that contamination by perchlorate is more widespread than previously thought.

Perchlorate is the dissociated anion of perchlorate salts such as potassium perchlorate, sodium perchlorate, and ammonium perchlorate and is extremely water soluble and environmentally stable. Therefore, the perchlorate ion is identical whether derived from potassium, sodium, or ammonium salts. Potassium perchlorate was used primarily as a pharmaceutical agent to treat hyperthyroidism. It is now used mainly in flares and automobile airbags, although it is still used for diagnostic purposes and for treatment of hyperthyroidism. Sodium perchlorate is used in the manufacture of slurry explosives. Ammonium perchlorate is widely used as a component of propellants for rockets, missiles, and fireworks (Soldin et al. 2001).

Perchlorate was first detected in high concentrations by monitoring wells in California during the early 1990s. When initially detected in California, the Region 9 Office of the U.S. Environmental Protection Agency developed a provisional reference dose for perchlorate of 0.0001 mg/kg/day in 1992. The reference dose later was revised to 0.0005 mg/kg/day in 1995. These values were based on a dated acute exposure study that showed that single doses of potassium perchlorate caused release of iodide from thyroids of patients with Graves disease, an autoimmune condition that results in hyperthyroidism (Stanbury and Wyngaarden 1952). In 2005 the reference dose was changed again to 0.0007 mg/kg/day.

## Mode of Action of Perchlorate in Humans

Perchlorate is a competitive inhibitor of the sodium iodide symporter (NIS), the thyroid cell–surface protein responsible for transporting iodide from the plasma into the thyroid. Therefore, it prevents further synthesis of the thyroid hormone (TH). It has no effect on the iodination process itself; rather, it displaces iodide by competitive uptake at the NIS. Perchlorate is concentrated by the thyroid tissue in a manner similar to iodide, but it is not significantly metabolized either in the gland or peripherally (Wolff 1998). Eskandari et al. (1997) disputed the notion that perchlorate is translocated via NIS into the cell and that perchlorate acts on NIS as a blocker, not as a substrate. Therefore, it is possible that perchlorate may cross the thyrocyte membrane by diffusion. In rats and humans, perchlorate appears to be eliminated rapidly, primarily in urine (>90%), virtually unchanged (Anbar et al. 1959; Eichler and Hackenthal 1962).

Several other inorganic anions such as thiocyanate and nitrate that are present in dietary and environmental sources have goitrogenic effects (Greer et al. 1966). Similar to perchlorate, they both competitively inhibit iodide uptake at NIS. Several studies have been conducted to determine the relative effects of perchlorate, thiocyanate, and nitrate on radioactive iodine uptake (RAIU) inhibition. Studies in rats showed that perchlorate was approximately 10 times more potent than thiocyanate and about 300 times more potent than nitrate in inhibiting RAIU in the thyroid. Furthermore, thiocyanate was slightly more potent than iodide (Wyngaarden et al. 1953). Tonacchera et al. (2004) demonstrated, in Chinese hamster ovary (CHO) cell lines stably transfected with the human *NIS* gene, that the relative potency of perchlorate on RAIU inhibition was 15, 30, and 240 times that of thiocyanate, iodide, and nitrate, respectively. The inhibiting effects when the cell lines where exposed to a mixture of perchlorate, thiocyanate, and nitrate were simply additive.

## Thyroid Hormone Synthesis

Thyroid hormone plays a key role in the growth and differentiation of many organs. It is especially important for development of the central nervous system during the prenatal and postnatal periods (reviewed by Zoeller et al. 2002). A severe shortage of TH for several weeks after birth results in serious mental and motor handicaps. During pregnancy the mother provides substantial amounts of TH to the fetus (Vulsma et al. 1989), so the delay in cerebral development caused by congenital hypothyroidism (CH) results mainly from postnatal TH deficiency. The risk for mental retardation and the difficulty in recognizing the disease were reasons for introducing neonatal mass screening programs. Therefore, the most serious effects of perchlorate might occur during the first trimester when the brain is forming and developing and TH supply is totally dependent on maternal supply of iodine and of thyroxine (T_4_) and triiodothyronine (T_3_)

To understand the potential impact of perchlorate on a gene–environment interaction model, we need to consider T_3_ and T_4_ in a proper biosynthesis context. TH synthesis and secretion are exquisitely regulated negative-feedback systems that involve the hypothalamus, pituitary, and thyroid glands. The hypothalamus secretes thyrotropin-releasing hormone (TRH), a tripeptide (pyroGlu-His-Pro) synthesized in the paraventricular nucleus of the hypothalamus. The TRH, transported by axons, binds to TRH receptors in the pituitary thyrotropes, a subpopulation of pituitary cells that secrete thyroid-stimulating hormone (TSH). TRH stimulation leads to release and synthesis of new TSH in thyrotropes. The TSH binds to the TSH receptor in the thyroid gland cells. TSH is the primary regulator of TH release and secretion. Both TRH and TSH secretion are negatively regulated by THs: when T_4_ reaches an adequate circulating level, the hypothalamus and pituitary reduce their output of TRH and TSH; they increase their output of TRH and TSH when the circulating blood level of T_4_ is low. A number of thyroid genes, including *NIS*, thyroglobulin (*Tg*), and thyroid peroxidase (*TPO*), are stimulated by TSH and promote the synthesis of TH (Zoeller 2003).

Iodine is critical to thyroid gland function and TH synthesis and secretion. The first step in thyroidal iodine metabolism is the cellular uptake of iodide from the extracellular fluid. The thyroidal iodine uptake is tightly regulated by the NIS, an intrinsic plasma membrane protein in the thyroid follicular cells (Dohán et al. 2003). From the follicular cell, the iodide moves across the apical membrane, transported by pendrin protein (Yoshida et al. 2002). The iodide is then delivered to the cell–colloid interface, where it is oxidized by TPO and bound to tyrosyl residues in the Tg. This iodination of specific tyrosines on Tg yields monoiodinated and diiodinated residues [monoiodotyrosines (MITs) and diiodotyrosines (DITs)] that are enzymatically coupled to form T_4_ and T_3_. The iodinated Tg containing MIT, DIT, T_4_, and T_3_ then is stored as an extracellular storage polypeptide in the colloid within the lumen of thyroid follicular cells.

Perchlorate does not undergo metabolism, but genetic defects of its target, that is, the NIS, may lead to low iodine uptake in the thyroid gland, thus depressing production of THs. In this scenario, exposure to perchlorate may further reduce the already low iodide uptake and decrease production of THs. The combined effects of perchlorate with a genetic decrease in THs would hence delineate a population at risk for decreased thyroid function.

We reviewed published data to identify genetic factors that might lead to different responses in people exposed to perchlorate in the environment. Because perchlorate inhibits iodide uptake, we focused on the genetic defects causing CH involving the iodination process of the THs, particularly *a*) defects in iodide transport from circulation into the thyroid cell; *b*) defects in iodide transport from the thyroid cell to the follicular lumen, often combined with inner ear deafness [Pendred syndrome (PDS)]; and *c*) defects of iodide organification.

A positive perchlorate discharge test is used as a diagnostic tool in most of these medical conditions. A positive diagnosis can be obtained by administering 1 g potassium perchlorate 2 hr after a tracer dose of ^131^I. In normal individuals, radioiodide accumulation in the thyroid gland ends after the administration of potassium perchlorate, but there is little loss of the thyroidal radioactivity previously accumulated in the gland. Instead, potassium perchlorate causes almost complete discharge of the unbound fraction of thyroid iodide in individuals with defects of iodide organification and with PDS. Therefore, these people could have different responses to environmental perchlorate exposure than normal individuals.

### Defects in iodide transport from circulation into the thyroid cell.

The NIS is the plasma membrane glycoprotein that mediates active iodide uptake into the thyroid follicular cells. This process is the crucial first step in TH biosynthesis. NIS couples the inward transport of sodium, which occurs in favor of its electrochemical gradient, to the simultaneous inward translocation of iodide against its electrochemical gradient. Two sodium ions per iodide ion are translocated into the cells (Dai et al. 1996; Eskandari et al. 1997). The sodium gradient that drives iodide uptake is maintained by the Na^+^/K^+^ ATPase.

Congenital iodide transport deficit (ITD) is an infrequent autosomic recessive condition characterized by inability of the thyroid gland to maintain a concentration gradient of iodide between the plasma and the thyroid follicular cell, resulting in hypothyroidism, diffuse or nodular goiter, and little or no uptake of radioiodine. The disorder has been linked to a defect of the NIS. In the absence of a functional NIS molecule, iodide has no access to the thyroid follicular cells, resulting in decreased TH biosynthesis and higher circulating levels of TSH, which in turn stimulates the morphologic and biochemical changes in the thyroid that result in development of goiter (De La Vieja et al. 2000).

The gene coding for human NIS has been mapped to chromosome 9p12-13.2. It has 15 exons and coding for a glycoprotein of 643 amino acids. NIS is a protein with 13 putative transmembrane domains, an extracellular amino terminus, and an intracellular carboxyl terminus (De La Vieja et al. 2000). About 58 cases of ITD from 33 families have been reported worldwide. Thirty of 31 cases from 21 families were studied at the molecular level and had several homozygous or compound heterozygous mutations of the perchlorate-sensitive *NIS* gene. Eleven mutations have been identified: V59E, G93R, Q267E, C272X, T354P, G395R, frameshift 515X, Y531X, G543E, ΔM142-Q323, and ΔA439-P443 (Fujiwara et al. 1997, 1998, 2000; Kosugi et al. 1998a, 1998b, 1999, 2002; Matsuda and Kosugi 1997; Pohlenz et al. 1997, 1998; Tonacchera et al. 2003). The single substitution in codon 354 converting from ACA (Thr) to CCA (Pro) was the most common mutation detected in 10 patients with homozygous mutations, and in four patients with compound heterozygous mutation (Fujiwara et al. 1997, 1998; Kosugi et al. 1998a, 1998b; Matsuda and Kosugi 1997). All were Japanese, suggesting that the mutant NIS T354P is more common in Japan. However, the frequency of this gene in the Japanese population is unknown because only 185 healthy people, representing only 370 alleles, have been genotyped.

The frequency of mutations in the *NIS* gene in the population is not known. Heterozygous persons do not express the phenotype; therefore, *NIS* gene defects can be detected only when both alleles are affected. People with homozygous mutations that cause partial loss of function may not be detected when, under conditions of high iodide intake, full preservation of iodide concentrating function is not required to achieve normal hormone synthesis. Therefore, impairment of thyroidal iodide concentration requires not only mutations in both *NIS* alleles but also defects that cause virtually complete loss of function.

The therapeutic treatment of ITD patients consists of l-T_4_ administration. Some patients also are supplemented with potassium iodide, thus underscoring the degree of functional loss of the mutated NIS. In these persons, perchlorate intake from contaminated sources could further reduce the functional activity of the mutated NIS in concentrating iodide in the thyroid.

### Defects in iodide transport from the thyroid cell to the follicular lumen, often combined with inner ear deafness (PDS).

PDS, an autosomal recessive disorder characterized by deafness and goiter, is the most common cause of syndromic deafness, accounting for up to 10% of all hereditary hearing loss (Fraser 1965; Nilsson et al. 1964). A phenotypic heterogeneity exists among affected persons, and thyroid dysfunction is particularly variable. At least 50% of affected persons have normal circulating levels of TH, whereas others develop clinical hypothyroidism (Reardon et al. 1999). Most affected persons demonstrate impaired iodide organification, as determined by a positive perchlorate discharge test. Hearing loss in PDS is prelingual and, in at least 80% of patients, is associated with structural defects of the inner ear, including a dilatation of the vestibular aqueduct and the Mondini defect of the cochlea (Johnsen et al. 1989). The PDS gene (*SLC26A4*) has been linked to chromosomal region 7q31 and contains an open reading frame of 2,343 bp encompassing 21 exons (Coyle et al. 1996; Sheffield et al. 1996). The predicted gene product pendrin is a highly hydrophobic 780 amino acid protein that transports chloride and iodide and mediates the exchange of chloride and formate. In the thyroid gland, a disorder in the function of pendrin may cause diminished iodide transport over the apical membrane that results in iodide remaining in the thyrocyte and a consequent decrease of organification of iodide. As a result, iodide accumulates in the cytoplasm and is discharged if thiocyanate or perchlorate is given (perchlorate discharge test). A decrease in the amount of radiolabeled iodide over the thyroid of > 10% is considered positive. At least 85 independent *SLC26A4* gene mutations have been characterized as causing PDS and nonsyndromic deafness, in some cases confirmed by a normal perchlorate discharge test (Adato et al. 2000; Blons et al. 2004; Bogazzi et al. 2000, 2004; Campbell et al. 2001; Coucke et al. 1999; Coyle et al. 1998; Everett et al. 1997; Fugazzola et al. 2000; Kopp et al. 1999; Li et al. 1998; Lopez-Bigas et al. 2002; Namba et al. 2001; Park et al. 2003; Prasad et al. 2004; Reardon et al. 2000; Scott et al. 2000; Tekin et al. 2003; Tsukamoto et al. 2003; Usami et al. 1999; Van Hauwe et al. 1998; Yong et al. 2001). Although these mutations are distributed throughout the coding sequence, having been identified in 19 of the 21 exons, the spectrum of mutations appears to show geographic differences. In Caucasian patients, the L236P, T416P, and IVS8+1G > A mutations account for nearly half of all *SLC26A4* mutant alleles, whereas in Japanese patients, these mutations are rare (Campbell et al. 2001; Tsukamoto et al. 2003). By contrast, H723R and ISV7–2A > G are the prevalent alleles accounting for most observed *SLC26A4* mutations in Korean and Japanese studies (Park et al. 2003; Tsukamoto et al. 2003). Some researchers have suggested that the frequency of these mutations could represent a founder effect rather than mutational hot spots.

A disorder in the function of pendrin will cause a diminished iodide transport over the apical membrane, which causes iodide to remain in the thyrocyte. Intake of perchlorate from a contaminated source may cause discharge of iodide from the thyrocyte, further exacerbating the organification defect, with resulting decrease of TH synthesis.

Moreover, at present, it is not known whether perchlorate will affect the function of the normal pendrin protein to transport iodide. Molecular studies addressing whether perchlorate may act on iodide transport through inhibition of the pendrin protein in a fashion similar to the NIS are needed and welcomed.

### Defects in iodide organification.

Iodide organification is the process by which iodine is oxidized and bound to thyrosine residue in Tg. Thyroid iodide organification disorder represents a group of defects characterized by discharge of substantial percentage of labeled iodide from the thyroid after administration of perchlorate (perchlorate discharge test) or thiocyanate. This discharge indicates a defect in converting accumulated iodide to organically bound iodine. The discharge may be partial or complete, thus defining partial or total defects. Partial iodide organification defects (PIODs) are characterized by release of < 50% of the accumulated radioiodine. Total iodide organification defects (TIODs) are characterized by release of > 90% of the accumulated radioiodine.

Iodination of the tyrosine residue is catalyzed by the membrane-bound thyroperoxidase (TPO). However, the oxidation of iodine requires hydrogen peroxide synthesized outside the thyroid follicular cell at the apical border catalyzed by the thyroid complex. Recently, two proteins of this complex, DUOX1 (also known as THOX1) and DUOX2 (also known as THOX2), have been identified (De Deken et al. 2000; Dupuy et al. 1999). The *DUOX1* and *DUOX2* genes are co-localized on the 15q15.3 chromosome and code for proteins of 1,551 and 1,548 amino acids, respectively. The DUOX1 and DUOX2 structure includes seven transmembrane-spanning domains, three NADPH- and one FAD-binding site, and 2EF-hand motifs. During the past three decades, few cases of thyroidal hydrogen peroxide have been described, but the molecular bases of these defects have just recently been investigated. Moreno et al. (2002) reported mutations in the *DUOX2* gene, resulting in premature stop codon, in four CH patients with unexplained iodide organification defects. One patient with permanent CH and TIOD carried a homozygous substitution, whereas three patients with temporary CH and PIOD carried heterozygous mutations that cause premature termination signal.

Lack of or insufficient activity of the DUOX2 protein diminishes hydrogen peroxide production, resulting in decreased activity of TPO and accumulation of iodide in the thyrocyte. Intake of environmental perchlorate, which inhibits iodine inflow, also may cause discharge of unbound iodine, further deteriorating the iodine organification process.

Under oxidative conditions, TPO catalyzes the coupling of iodotyrosines to iodothyronine residue in Tg. Thyroperoxidase is a glycosylated hemoprotein encoded by the *TPO* gene located on chromosome 2p25. The gene contains 17 exons coding for a protein of 933 amino acids. The protein has a transmembrane helix with a large extracellular N-terminal part containing a heme group. TPO defects are believed to be among the most frequent causes of abnormalities in thyroid iodide organification defect causing goitrous CH. TPO activity is not detectable in thyroid tissue of patients with TIOD. Absence of TPO activity implicates the inability to iodinate tyrosine residue in Tg and to couple these residues to form THs, mainly T_4_ and some T_3_ and rT_3_ (reverse T_3_) Inactivating mutations in both *TPO* alleles have been found in patients with CH caused by TIOD. With use of a variety of molecular techniques for mutation deletion, 36 mutations have now been defined for *TPO*. These include frameshift mutations caused by nucleotide insertion or deletion, as well as missense, nonsense, and splice site mutations (Abramowicz et al. 1992; Ambrugger et al. 2001; Bakker et al. 2000; Bikker et al. 1994, 1995, 1997; Kotani et al. 2001; Nascimento et al. 2003; Niu et al. 2002; Pannain et al. 1999; Rivolta et al. 2003; Santos et al. 1999; Umeki et al. 2002, 2004; Wu et al. 2002). The first reported mutation was a homozygous GGCC insertion in exon 8 of the *TPO* gene. The resulting frameshift generates a stop codon in exon 9, which results in a grossly truncated protein with no expected activity (Abramowicz et al. 1992). In a Dutch study of 45 patients from 40 families with CH caused by TIOD, the GGCC insertion in exon 8 at nucleotide position 1287 was the most common mutation found (Bakker et al. 2000). It was detected in 36% of the investigated *TPO* alleles and in 51% of the families investigated either in a homozygous or a compound heterozygous fashion. In this study, mutations in both *TPO* alleles were found in 29 families: for 13 families in a homozygous fashion and for 16 families in a compound heterozygous fashion. A total of 16 different mutations were found, including 8 novel mutations: 6 frame-shift mutations, 6 missense mutations, 3 splice site mutations, and 1 nonsense mutation. Most of these mutations occurred in exon 8, 9, or 10, which encode for the active part of the enzyme involved in the heme binding. In one patient with classic TIOD, a homozygous deletion in exon 14 appeared to have resulted from partial maternal isodisomy of the short arm of chromosome 2 carrying the defective *TPO* gene (Bakker et al. 2001). In some patients alternative splicing would generate a partially active form of the enzyme. In others an early termination signal would prevent translation of the fully active protein (Abramowicz et al. 1992; Bikker et al. 1994, 1995; Mangklabruks et al. 1991; Santos et al. 1999). Umeki et al. (2002) described 2 novel mutations in the *TPO* gene, R665W and G771R, in exons 11 and 13, respectively. The former was found in the patient’s father (heterozygous) and the latter in her mother, also heterozygous. No TPO activity was detectable with cells transfected with mutated mRNAs. Moreover, the mutated TPO proteins showed abnormal cellular localization, exhibiting immunofluorescence only in the intracellular structure. Therefore, the loss of apical membrane localization of the mutated TPO was the main cause for the iodide organification defect.

PIODs also can be caused by disorders in TPO. In an investigation of *TPO* mutations in five families with PIOD, Nascimento et al. (2003) found a compound heterozygous mutation in three patients from one family inherited from both heterozygous parents. In the other four families, they found only heterozygous *TPO* mutations or polymorphisms, suggesting the translated protein could be partially inactive. Recently, PIOD caused by *TPO* gene was diagnosed in three siblings (Kotani et al. 2003). The three siblings with goiter and latent-to-mild hypothyroidism had a compound heterozygous mutation for a missense mutation (G1687T) and a deletion in exon 10 (1808-13del), resulting in a produced protein with two deleted amino acids ΔD574-L4575. From the expression studies, the mutated ΔD574-L4575–TPO synthesized THs to some extent (Kotani et al. 2003).

A common feature of patients with thyroid organification disorders syndrome is the discharge of iodine from the thyroid after administration of perchlorate. The level of perchlorate administrated in the diagnostic test is higher than the reported level of contaminated sources. However, it is biologically plausible that cumulative ingestion of perchlorate through a contaminated source may cause some degree of iodine discharge from thyrocytes. In populations with partial activity of the TPO enzyme, exposure to high enough levels of environmental perchlorate could cause unbound iodide discharge; therefore, less iodine will be available for biosynthesis of THs, thus further deteriorating their conditions.

## Relevant Studies of Perchlorate in Humans

Many studies have attempted to provide useful information on the dose–response relation of perchlorate-related health effects. Several ecologic studies have compared thyroid function in newborns using T_4_ and TSH screening data in infants born to mothers in areas with different perchlorate exposure. However, these studies yielded contradictory results. Brechner et al. (2000) found higher TSH in newborns in Yuma, Arizona, which has high perchlorate exposure, than in Flagstaff, Arizona, which has lower exposure. However, whether perchlorate exposure caused the observed TSH effect cannot be addressed because of the lack of direct perchlorate measurement in the study. By contrast, F.X. Li et al. (2000) and Z. Li et al. (2000) found no association in Nevada newborns between low T_4_ and TSH levels and perchlorate exposure. A limitation of these studies is that the investigators did not collect data on individual exposure to perchlorate and on iodine intake levels. In a population-based ecologic study using California Newborn Screening Program data, Schwartz (2001) claimed to identify a significant dose–response association between perchlorate exposure and T_4_, and an association of perchlorate exposure and being a presumptive positive for CH. These data contrast with a previous ecologic analysis (Lamm and Doemland 1999) that found no increase of CH incidence in California and Nevada counties with perchlorate levels of 4–16 μg/L in drinking water supplies.

Crump et al. (2000) conducted a study in three proximate cities in northern Chile that had different concentrations of perchlorate in tap water, involving 162 school-age children and 9,784 newborns. These authors found no alteration of thyroid function or incidence of CH in Taltal, Chile, where the tap water contained 100–120 μg/L perchlorate, compared with two other regions of Chile with low or no perchlorate in the water. However, the data also showed high levels of urine iodine, indicating that iodine intake in the population was very high, possibly overcoming the inhibitory effect of perchlorate on thyroid function.

To establish the dose response in humans for the perchlorate inhibition of thyroidal iodide uptake and the short-term effects on circulating TH, Greer et al. (2002) gave perchlorate in drinking water at 0.007, 0.02, 0.1, or 0.5 mg/kg per day to 37 male and female volunteers for 14 days. In 24 participants 8-and 24-hr measurements of thyroidal ^123^I uptake (RAIU) were performed before exposure, on exposure days 2 and 14, and 15 days postexposure. Results from the study indicated a true no-effect level of perchlorate of 5.2 or 6.4 μg/kg/day for RAIU. Considering that a 70 kg adult drinks 2 L of water per day, this dose would be ingested if the drinking water contained 182–224 μg/L. In addition, the dose of 0.5 mg/kg/day taken for 14 days did not produce changes in circulating levels of T_4_ or TSH, suggesting that short-term consumption of perchlorate levels of 17.5 mg/L in drinking water would not affect circulating levels of THs. The authors suggested that this failure of perchlorate to influence circulating levels of TH resulted from the storage capacity of the normal adult thyroid gland, which contains unreleased stored hormones lasting for several months. However, as pointed out by Zoeller (2003), the case may be different for a late gestation fetus or neonate, where the estimated intrathyroidal amount of hormone stored is less than that required for 1 day (Van den Hove et al. 1999; Vulsma et al. 1989). Thus, the concentration of perchlorate sufficient to reduce thyroidal iodine uptake in a fetus or neonate may be sufficient to produce a significant decrement in circulating levels of TH. The fetal thyroid gland obtains iodide for its own TH synthesis from the maternal circulation through the placenta. Placental transfer of perchlorate has been reported in guinea pig (Postel 1957). In human, whether perchlorate crosses from the mother to the fetus during pregnancy is not known. However, this placental transfer could be biologically plausible because expression of the *NIS* has been reported in human placenta (Bidart et al. 2000). Moreover, perchlorate may concentrate in milk because the NIS protein is induced in lactating breast tissue by prolactin (Tazebay et al. 2000). Perchlorate might decrease iodide uptake into milk, thus reducing the sole source of iodine to the infant. Differently from adults, who most likely can recover from transient hypothyroidism without permanent health consequences, a short period of TH insufficiency may produce permanent neurologic deficits in children (Van Vliet 1999). The study of no-effect level (Greer et al. 2002) was conducted in healthy adults with normal iodine intake, and it is debatable whether 14 days is sufficient time to illustrate perchlorate effect on humans. This no-effect level most likely would be lower in populations with genetic defects causing CH and in populations with lower iodine uptake. The Third National Health and Nutrition Examination Survey (NHANES III), conducted during 1988–1994, found that the percentages of males and females with urinary iodine concentrations < 5 μg/dL were substantially higher in every age category than in the 1971–1974 survey (Hollowell et al. 1998). In pregnant women, these percentages were 6.9% in NHANES III and 1.0% in NHANES I (Hollowell et al. 1998). The overall decline in the last few decades raises concern that a fairly large number of people in the United States may lack adequate iodine intake.

## Conclusions

Exposure to perchlorate, which inhibits iodine uptake, has the biologic potential to cause hypothyroidism and, in pregnant women, severely damage the fetus and the newborn. NHANES III data suggest that 4.3% of the U.S. population may be subclinically hypothyroid (Hollowell et al. 2002). CH affects about 1 in 3,000 to 1 in 4,000 infants and in about 15% of cases may result from a defect of thyroid hormonogenesis, mostly inherited in an autosomal recessive fashion (Vulsma and de Vijlder 2000). Such defects may result from abnormalities in several steps involved in TH synthesis. Our literature review identified possible homozygous or compound heterozygous mutations of genes involved in thyroid iodine synthesis that cause hypothyroidism that could be used to define a potential susceptible population to perchlorate exposure. In a Mendelian fashion, the number of carriers of heterozygous mutated gene causing CH would be higher than the number of the reported CH cases. Given the logical connection between perchlorate, diminished iodine uptake, hypothyroidism, and thyroid-related health effects, people exhibiting heterozygous or homozygous genetic mutations in genes involved in the TH synthesis, especially in a milieu of low iodine uptake, can reasonably be expected to be more susceptible than people who show no genetic variability to the effects of perchlorate. Several studies based on T_4_ and TSH screening data in infants born to mothers in areas with different perchlorate exposure mostly have found no increase in hypothyroidism incidence. However, these studies lacked estimates of individual perchlorate exposure, as well as estimates of individual iodine uptake. The only study that included iodine values showed no significant association between perchlorate and hypothyroidism. However, it showed high urinary iodide, suggesting the high iodine uptake could easily have upset the inhibition factor of the perchlorate. We conclude that future epidemiologic and population-based studies as well as no-effect studies concerning the link between human disease and environmental perchlorate exposure should consider among their variables the genetic makeup of the participants, actual perchlorate exposure levels, and individual iodine uptake and excretion levels.
